# 

**Published:** 1959-04

**Authors:** 


### Contents

| | | Page |
|---|---|---|
| A REVIEW OF ACUTE INTESTINAL OBSTRUCTION | *Mr. P. J. W. Monks* | 31 |
| SQUINT | *Dr. Calbert I. Phillips* | 40 |
| TRANSPORTING SEVERELY PARALYSED PATIENTS | *Dr. James Macrae* | 45 |
| APLASTIC ANAEMIA WITH POST-TRANSFUSIONAL HAEMOSIDEROSIS | *Clinical Pathological Conference* | 47 |
| NOTES AND NEWS | | 51 |
| APPOINTMENTS | | 51 |

